# Inbreeding depression does not increase after exposure to a stressful environment: a test using compensatory growth

**DOI:** 10.1186/s12862-016-0640-1

**Published:** 2016-04-01

**Authors:** Regina Vega-Trejo, Megan L. Head, Michael D. Jennions

**Affiliations:** Division of Evolution, Ecology and Genetics, Research School of Biology, The Australian National University, Acton, ACT 2601 Australia

**Keywords:** Fitness, Food stress, Catch-up growth, Growth rates, Mosquitofish

## Abstract

**Background:**

Inbreeding is often associated with a decrease in offspring fitness (‘inbreeding depression’). Moreover, it is generally assumed that the negative effects of inbreeding are exacerbated in stressful environments. This G × E interaction has been explored in many taxa under different environmental conditions. These studies usually manipulate environmental conditions either in adulthood or throughout an individual’s entire life. Far fewer studies have tested how stressful environments only experienced during development subsequently influence the effects of inbreeding on adult traits.

**Results:**

We experimentally manipulated the diet (control versus low food) of inbred and outbred juvenile Eastern mosquitofish (*Gambusia holbrooki*) for three weeks (days 7-28) to test whether experiencing a presumably stressful environment early in life influences their subsequent growth and adult phenotypes. The control diet was a standard laboratory food regime, while fish on the low food diet received less than 25 % of this amount of food. Unexpectedly, despite a large sample size (237 families, 908 offspring) and a quantified 23 % reduction in genome-wide heterozygosity in inbred offspring from matings between full-siblings (*f* = 0.25), neither inbreeding nor its interaction with early diet affected growth trajectories, juvenile survival or adult size. Individuals did not mitigate a poor start in life by showing ‘compensatory growth’ (i.e. faster growth once the low food treatment ended), but they showed ‘catch-up growth’ by delaying maturation. There was, however, no effect of inbreeding on the extent of catch-up growth.

**Conclusions:**

There were no detectable effects of inbreeding on growth or adult size, even on a low food diet that should elevate inbreeding depression. Thus, the long-term costs of inbreeding due to lower male reproductive success we have shown in another study appear to be unrelated to inbreeding depression for adult male size or the growth rates that are reported in the current study.

**Electronic supplementary material:**

The online version of this article (doi:10.1186/s12862-016-0640-1) contains supplementary material, which is available to authorized users.

## Background

Mating with relatives occurs commonly in small populations and can result in a decline in offspring performance (ideally measured as fitness) known as inbreeding depression [1]. Inbreeding depression typically has important consequences for variation in lifetime fitness and juvenile development both within and among populations [1, 2]. Due to an increase in homozygosity, inbreeding can reduce performance by either decreasing the frequency of heterozygotes (overdominance) or unmasking deleterious recessive alleles (partial dominance; [3]). Regardless of the mechanism by which inbreeding depression arises, it is usually more readily detected in traits that are linked with fitness (e.g. key life history traits such as growth rates, size at adulthood, and juvenile survival; [4–7]). This is because strong directional selection promotes fixation of advantageous genes, which means that traits linked with fitness have a higher proportion of dominance relative to additive genetic variance [8–10]. Many studies show that inbreeding affects individual traits (e.g. life history, morphology, physiology, and behaviour; [11, 12]). Even so, our understanding of what factors cause variation in the extent to which inbreeding has deleterious effects, and why some traits are affected but not others, remains limited.

The extent of inbreeding depression may be affected by the environment an individual experiences [13]. Stressful environments (i.e. environments that reduce fitness relative to other environments; [14]) are generally expected to exacerbate the effects of inbreeding [1, 14, 15]. However, over a broad range of taxa and conditions, studies looking at the interaction between inbreeding and stressful conditions have yielded inconsistent results [16–18]. Different species, populations, inbred lines, sexes, and families are highly variable in their response to inbreeding and different types of stress [18–20]. An extensive review by Armbruster et al. [14] found that inbreeding depression increased by 69 % on average in stressful environments, but increased significantly in fewer than half the studies. More recently, a meta-analysis has suggested that the effect of the environment on inbreeding scales linearly with the magnitude of the stress imposed [16]. Thus it appears that the level and type of stress experienced play some part in explaining variation in the severity of inbreeding depression.

A further explanation for the inconsistent effects that stressful environments have on inbreeding depression is that it depends on the developmental or life history stage at which stress is experienced [21–23]. However, most studies look at how stressful environments experienced during adulthood or throughout an organism’s life influence the effects of inbreeding [14, 16]. Relatively few studies investigate how stressful environments experienced during particular life stages and, more specifically, during early-life affect the subsequent performance of inbred and outbred individuals [13, 24]. Only six studies in a major review by Fox and Reed [16] explored the interaction between inbreeding and an environmental stress that was restricted to early in life.

A restricted diet during development has the potential to reduce adult body size and consequently lower fecundity, increase predation, and reduce mating success, among other costs [25–29]. Given the potential fitness costs of small adult body size, animals often respond to periods of diet restriction during their juvenile growth phase by increasing growth rates once their diet returns to normal (‘compensatory growth’) or by delaying maturity until they reach a normal size (‘catch-up growth’; meta-analysis: [30]). However, these responses often incur costs such as increased predation risk, changes in locomotor performance, and a reduced lifespan (see [28] for a review). The lack of studies that explore the relationship between inbreeding and a dietary stress early in life is unexpected given the burgeoning interest in ‘compensatory growth’ to make up for a ‘poor start’ in life (reviews: [29, 30]) with putative long term costs of elevated ‘catch-up’ growth [31, 32]. To date, there are surprisingly few experimental studies documenting levels of inbreeding depression that use restricted food availability early in life as an environmental stress and measure its effects on growth and any carry-over effects on size at maturity or other adult traits (but see [4, 33–36]). It is reasonable to assume that the ability to respond to a restricted diet during early development will depend on genotype (e.g. level of heterozygosity, additive genetic variation for fitness; [4, 28]), including the decline in heterozygosity that arises with inbreeding.

Here, we manipulate the amount of food given to experimentally create inbred (F_1_ offspring of matings between full siblings, *f* = 0.25) and outbred (F_1_ offspring of unrelated parents) juvenile Eastern mosquitofish (*Gambusia holbrooki*). Fish in the control treatment received the standard laboratory diet, while those on a low food treatment received less than 25 % of this amount of food for a 21-day period during early development (days 7-28 after birth) before returning to the control diet. We used data from over 3000 SNPs to confirm that inbreeding reduced genome-wide heterozygosity. We then quantified the interaction between inbreeding and experiencing a presumably more stressful rearing environment. Specifically, we aim to test whether diet restriction during early development differentially influences subsequent growth trajectories and adult phenotype depending on whether an individual is inbred or outbred.

Previous work has shown that female, but not male, *G. holbrooki* show compensatory growth when assigned to our low food treatment, and that both sexes exhibit catch-up growth, albeit with a proportionately longer delay in maturation time for males than females [37]. In addition, we have shown that males reared on the low food treatment are less attractive to females [38]. This suggests that they are less fit so, by definition (sensu [14]), the low food treatment is ‘stressful’.

To date there have been almost no studies experimentally manipulating inbreeding in *G. holbrooki* (but see [39]). More generally, however, there is good evidence that inbreeding lowers a range of performance measures in another poeciliid fish, the guppy (e.g. fecundity [40], male reproductive performance [41], sperm number [42, 43], clutch size, and survival [44, 45]). We did, however, use a subset of the current data [46] to show that there is no effect of inbreeding on size at birth and growth over the first seven days in *G. holbrooki*. There is, however, a decline in brood size suggestive of inbreeding elevating embryo mortality. More importantly, we have recently shown that the inbred sons of full-siblings gain a lower share of paternity when they compete with outbred males (Vega-Trejo, R, Head ML, Keogh SJ, Jennions MD unpublished observations). Finally, Kruuk et al. [47] recently reported consistent variation among families in their growth rate on control and low food diets. Given inbreeding generally lowers performance it seems worthwhile to test whether the more ‘extreme’ genotypes created by inbreeding extend the genetic variation beyond that naturally occurring which might then explain some of the variation in growth patterns.

Given these previous studies we predict that:

- Inbred fish will generally have slower growth rates, take longer to mature, and be smaller at adulthood than outbred fish (i.e. inbreeding depression for growth and size).
- Inbreeding depression will be greater when fish are placed on a restricted diet as juveniles (i.e. a G × E interaction between inbreeding and diet).
- Inbred fish will show weaker compensatory and/or catch-up growth than outbred individuals (i.e. this is the mechanism generating the G × E interaction).

## Results

### Inbreeding and heterozygosity

We confirmed that there is sufficient genetic variation in our study population for a full-sibling mating to have a readily detectable effect on offspring heterozygosity. Based on data from over 3000 SNP loci, we found that a brother-sister mating led to a significant decline in offspring heterozygosity (F_(1,120)_ = 215.1, *P* < 0.001). The mean heterozygosity of inbred fish was 23.2 % less than that of outbred fish (very close to a 25 % decline, which is the expected reduction in heterozygosity due to a full-sib mating in an outbred population). The proportion of loci that were heterozygous was 0.239 ± 0.003 in inbred males (*n* = 62) and 0.311 ± 0.004 in outbred males (*n* = 62). Hereafter we therefore use inbred versus outbred status in our analysis.

### Is there an effect of inbreeding on mosquitofish?

Contrary to our predictions, we did not find any evidence of inbreeding depression. This was the case in both the control environment, and in the stressful low food environment that led to almost zero growth over the three-week period in which food was restricted (see below). We have previously reported the effects of inbreeding on birth size and growth to 7 days using a subset of the current data [46]. With the current larger dataset we still found no difference in size at birth, or size at one week of age (before the diet treatment was imposed) between inbred and outbred fish (see also [46]). We also found no significant effect of inbreeding on growth rates, adult size, age at maturity, survival until adulthood, or the sex ratio at maturity (Tables 1 and 2).

**Table 1:** Results from mixed models with chi squares (χ^2^) values for significance tests of estimated parameters for inbreeding and food treatment

Response variable	*N*	Predictor	Estimate	SE	χ^2^	*P*
Length at birth [ln(mm)]	1221	Intercept	0.869	0.002	47498.302	**<0.001**
		Inbreeding (inbred)	3.52 × 10^-4^	2.64 × 10^-3^	0.046	0.892
Growth day 0 – day 7 (ln[mm]/day)	OM: 234 IM: 241 OF: 233 IF: 200	Intercept	0.057	5.2 × 10^-4^	11701.432	**<0.001**
		Inbreeding (outbred)	6.2 × 10^-4^	4.0 × 10^-4^	2.355	0.125
		Sex (male)	-5.9 × 10^-4^	2.5 × 10^-4^	5.456	**0.020**
		Inbreeding × Sex	3.1 × 10^-4^	2.5 × 10^-4^	1.510	0.220
Growth day 7 – day 28 (ln[mm]/day)	OM: 234	Intercept	1.4 × 10^-2^	1.15 × 10^-4^	16580.458	**<0.001**
	IM: 241					
	OF: 233					
	IF: 200					
		Inbreeding (outbred)	7.6 × 10^-5^	9.6 × 10^-5^	0.616	0.432
		Diet (control)	1.1 × 10^-2^	8.1 × 10^-5^	21098.343	**<0.001**
		Sex (male)	-2.4 × 10^-4^	8.2 × 10^-5^	8.684	**0.003**
		Inbreeding × Diet	-8.7 × 10^-5^	8.1 × 10^-5^	1.156	0.282
		Diet × Sex	-4.0 × 10^-4^	8.3 × 10^-5^	23.766	**<0.001**
		Inbreeding × Sex	-5.5 × 10^-5^	8.3 × 10^-5^	0.447	0.503
		Inbreeding × Diet × Sex	8.9 × 10^-5^	8.3 × 10^-5^	1.143	0.284
Growth day 28 – day 49 (ln[mm]/day)	OM: 234	Intercept	1.3 × 10^-2^	2.2 × 10^-4^	3666.595	**<0.001**
	IM: 241					
	OF: 233					
	IF: 200					
		Inbreeding (outbred)	7.2 × 10^-5^	1.7 × 10^-4^	0.177	0.673
		Diet (control)	-7.6 × 10^-3^	9.1 × 10^-5^	6939.440	**<0.001**
		Sex (male)	-3.8 × 10^-4^	9.6 × 10^-5^	16.263	**<0.001**
		Inbreeding × Diet	2.8 × 10^-5^	9.1 × 10^-5^	0.097	0.756
		Diet × Sex	1.8 × 10^-4^	9.6 × 10^-5^	3.510	0.061
		Inbreeding × Sex	-4.6 × 10^-5^	9.6 × 10^-5^	0.229	0.632
		Inbreeding × Diet × Sex	-6.9 × 10^-5^	9.6 × 10^-5^	0.514	0.474
Initial compensatory growth—Growth control diet (7-28) vs low food diet (28-49) (ln[mm]/day)	OM: 234	Intercept	2.4 × 10^-2^	1.8 × 10^-4^	16803.581	**<0.001**
	IM: 241					
	OF: 233					
	IF: 200					
		Inbreeding (outbred)	1.3 × 10^-5^	1.4 × 10^-4^	0.009	0.9262
		Diet (control)	2.5 × 10^-3^	1.0 × 10^-4^	600.251	**<0.001**
		Sex (male)	-5.4 × 10^-4^	1.0 × 10^-4^	26.422	**<0.001**
		Inbreeding × Diet	-8.9 × 10^-6^	1.0 × 10^-4^	0.008	0.9305
		Diet × Sex	-3.7 × 10^-5^	1.0 × 10^-4^	0.126	0.7227
		Inbreeding × Sex	6.4 × 10^-5^	1.0 × 10^-4^	0.368	0.544
		Inbreeding × Diet × Sex	1.7 × 10^-5^	1.0 × 10^-4^	0.028	0.868
Overall compensatory growth—Growth from 7 (control diet) or 28 (low food diet) to sexual maturity (ln[mm]/day)	OM: 233	Intercept	0.041	0.001	1542.2322	**<0.001**
	IM: 241					
	OF: 233					
	IF: 198					
		Inbreeding (outbred)	8.9 × 10^-4^	6.2 × 10^-4^	2.036	0.154
		Diet (control)	-1.6 × 10^-4^	5.3 × 10^-4^	0.087	0.768
		Sex (male)	-1.5 × 10^-4^	5.4 × 10^-4^	0.074	0.786
		Inbreeding × Diet	-3.1 × 10^-4^	5.3 × 10^-4^	0.346	0.556
		Diet × Sex	2.7 × 10^-4^	5.5 × 10^-4^	0.248	0.619
		Inbreeding × Sex	5.6 × 10^-4^	5.5 × 10^-4^	1.066	0.302
		Inbreeding × Diet × Sex	3.4 × 10^-4^	5.5 × 10^-4^	0.524	0.469
Catch-up growth—Length at maturity [ln(mm)]	OM: 233	Intercept	1.364	1.8 × 10^-3^	5.3 × 10^-5^	**<0.001**
	IM: 241					
	OF: 233					
	IF: 199					
		Inbreeding (outbred)	-1.0 × 10^-3^	1.4 × 10^-3^	0.484	0.487
		Diet (control)	5.7 × 10^-3^	1.2 × 10^-3^	21.57	**<0.001**
		Sex (male)	-2.1 × 10^-3^	1.2 × 10^-3^	2.94	0.086
		Inbreeding × Diet	-4.2 × 10^-5^	1.2 × 10^-3^	1.2 × 10^-3^	0.972
		Diet × Sex	-2.8 × 10^-3^	1.2 × 10^-3^	5.019	**0.025**
		Inbreeding × Sex	5.5 × 10^-5^	1.2 × 10^-3^	2.0 × 10^-3^	0.964
		Inbreeding × Diet × Sex	8.5 × 10^-4^	1.2 × 10^-3^	0.460	0.498
Catch-up growth—Age at sexual maturity [ln(days)]	OM: 233	Intercept	4.501	0.023	39313.078	**<0.001**
	IM: 241					
	OF: 233					
	IF: 199					
		Inbreeding (outbred)	-0.016	0.016	1.014	0.314
		Diet (control)	-0.131	0.013	107.673	**<0.001**
		Sex (male)	-0.031	0.012	5.723	**0.017**
		Inbreeding × Diet	0.013	0.012	1.001	0.317
		Diet × Sex	0.009	0.013	0.477	0.489
		Inbreeding × Sex	0.018	0.013	1.979	0.159
		Inbreeding × Diet × Sex	0.005	0.013	0.154	0.694
Survival from day of birth to maturity		Intercept	20.217	177.037	0.013	0.909
		Inbreeding (outbred)	0.064	175.037	0	0.999
		Diet (control)	-0.023	192.792	0	0.999
		Sex (male)	-0.052	180.058	0	0.999
		Inbreeding × Diet	5.144	177.420	8 × 10^-4^	0.977
		Diet × Sex	5.172	177.257	9 × 10^-4^	0.977
		Inbreeding × Sex	-5.234	177.075	9 × 10^-4^	0.976
		Inbreeding × Diet × Sex	-0.027	178.041	0	0.999
Offspring sex ratio (proportion male)		Intercept	-0.096	0.067	2.058	0.151
		Inbreeding (outbred)	0.091	0.067	1.882	0.170
		Diet (control)	-0.032	0.067	0.238	0.626
		Inbreeding × Diet	0.037	0.067	0.303	0.582

Numbers in bold indicate significant values. *OM* outbred males, *IM* inbred males, *OF* outbred females, *IF* inbred females. N varied in the analysis due to individuals not being measured at adulthood or died

**Table 2:** Means and SE from raw data separated by sex and food treatment

	Outbred		Inbred	
Length at birth (mm)	7.375 (0.017)		7.378 (0.016)	
	Outbred control diet	Inbred control diet	Outbred low food diet	Inbred low food diet
Male growth day 0 – day 7 (ln[mm]/day)	0.058 (0.0007)	0.057 (0.0007)	0.056 (0.0007)	0.055 (0.0008)
Male length at day 7 (mm)	11.101 (0.057)	11.005 (0.062)	10.974 (0.059)	11.017 (0.073)
Male growth day 7 – day 28 (ln[mm]/day)	0.026 (0.0002)	0.026 (0.0002)	0.003 (0.0001)	0.003 (0.0002)
Male length at day 28 (mm)	19.133 (0.090)	18.942 (0.096)	11.725 (0.079)	11.770 (0.092)
Male growth day 28 – day 49 (ln[mm]/day)	0.006 (0.0002)	0.006 (0.0002)	0.021 (0.0003)	0.021 (0.0003)
Male compensatory growth control diet (7-28) vs low food diet (28-49) (ln[mm]/day)	0.026 (0.0002)	0.026 (0.0002)	0.021 (0.0003)	0.021 (0.0003)
Male catch-up growth control diet (7-maturity) vs low food diet (28-maturity) (ln[mm]/day)	0.040 (0.002)	0.040 (0.001)	0.042 (0.002)	0.040 (0.002)
Male length at maturity (mm)	23.243 (0.175)	23.302 (0.173)	22.779 (0.135)	23.047 (0.139)
Male age at sexual maturity (days)	80.570 (3.471)	77.298 (2.817)	97.258 (3.368)	100.479 (3.398)
Female growth day 0 – day 7 (ln[mm]/day)	0.059 (0.0008)	0.058 (0.0008)	0.058 (0.0007)	0.058 (0.0008)
Female length at day 7 (mm)	11.145 (0.065)	11.124 (0.069)	11.162 (0.058)	11.084 (0.059)
Female growth day 7 – day 28 (ln[mm]/day)	0.027 (0.0003)	0.027 (0.0003)	0.003 (0.0002)	0.002 (0.0001)
Female length at day 28 (mm)	19.683 (0.123)	19.707 (0.127)	11.919 (0.078)	11.688 (0.078)
Female growth day 28 – day 49 (ln[mm]/day)	0.007 (0.0002)	0.006 (0.0003)	0.022 (0.0003)	0.022 (0.0003)
Female compensatory growth control diet (7-28) vs low food diet (28-49) (ln[mm]/day)	0.027 (0.0003)	0.027 (0.0003)	0.022 (0.0003)	0.022 (0.0004)
Female catch-up growth control diet (7-maturity) vs low food diet (28-maturity) (ln[mm]/day)	0.043 (0.001)	0.040 (0.001)	0.042 (0.002)	0.040 (0.002)
Female length at maturity (mm)	23.617 (0.203)	23.920 (0.215)	22.857 (0.211)	22.916 (0.246)
Female age at sexual maturity (days)	78.781 (3.964)	83.084 (3.926)	104.193 (4.146)	113.615 (5.096)
Survival	96.20 %	93.19 %	95.58 %	90.95 %
Sex ratio (M:F)	114:114	124:96	120:119	117:104

### Is inbreeding depression exacerbated under a stressful environment?

Contrary to our predictions, we did not find any evidence of an interaction between inbreeding and the diet treatment for any of the nine traits measured (Table 1). There is therefore no evidence that inbreeding depression for these traits is elevated after individuals are exposed to the more stressful low food environment.

### Does diet affect growth rate in mosquitofish?

Note, when testing for an effect of diet on growth rate we always included inbreeding status in the model. Prior to imposing the diets, we found a sex difference in growth from birth to one week of age due to females growing significantly faster (Table 1). Given that control diet fish were fed *ad libitum* with *A. nauplii* twice a day throughout the experiment and low food diet had their food restricted from 7 to 28 days of age when they were fed 3 mg of *A. nauplii* once every other day, we found a significant difference between fish on the control and low food diet in the mean growth rate from day 7 to day 28. As expected, the low food diet almost totally suppressed growth, resulting in far smaller fish by day 28. Females still grew significantly faster than males when fish were on the control diet, but not when on the low food diet, presumably because there was so little growth by either sex (Tables 1 and 2).

**Fig. 1:**
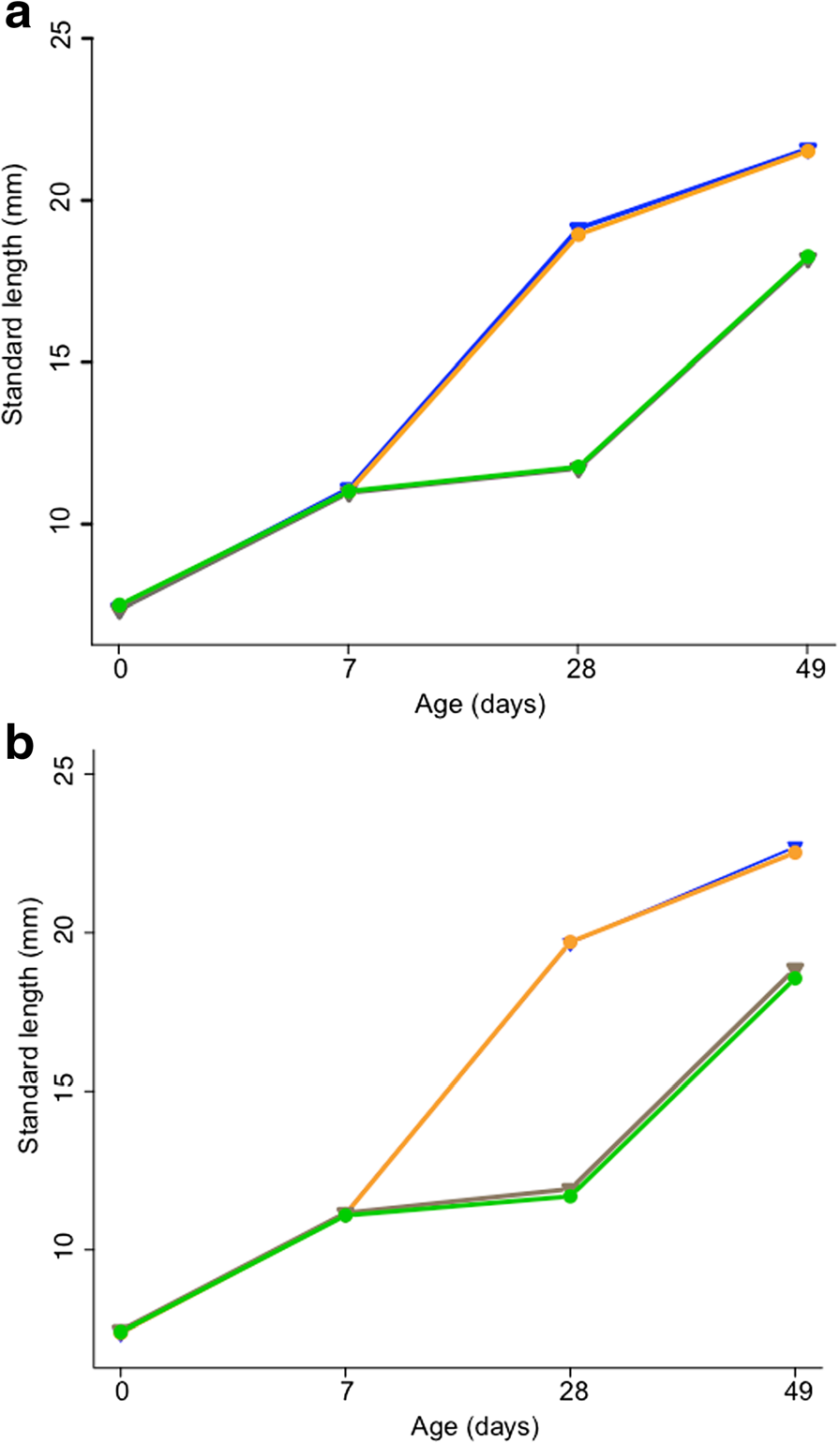
Mean growth trajectories of fish separated by inbreeding and diet. Data shown for growth periods prior to sexual maturity for (**a**) females, (**b**) males. Blue triangles = outbred control diet, brown triangles = outbred low food diet, orange circles = inbred control diet, green circles = low food diet

When fish on the low food diet were returned to the same diet as that of control fish, they showed a significant increase in growth from day 28 to 49 compared to control fish. This was, however, due to their smaller size at the beginning of this period. We did not find any evidence of initial compensatory growth when comparing growth from a comparable starting size (Fig. 1). Although fish on each diet had a similar starting size (that is, growth from day 7 – 28 for control diet and growth from day 28-49 for low food diet fish; Table 2), those on the low food diet actually showed significantly slower growth immediately after returning to a normal diet. In general, after day 28 (the end of the low food diet), females grew significantly faster than males regardless of diet treatment. We did not find any evidence for overall compensatory growth; growth to sexual maturity was not affected by diet nor did it differ between the sexes.

**Fig. 2:**
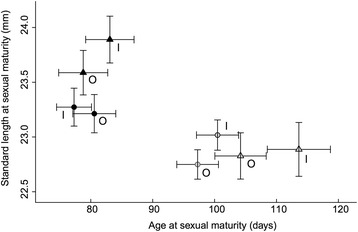
Mean age and length at sexual maturity and 95 % confidence interval for fish separated by inbreeding, diet, and sex. O = outbred, I = inbred, triangles = females, circles = males, black = control diet, grey = low food diet. Outbred control males *N* = 114, Outbred control females *N* = 114, Outbred low food diet males *N* = 119, Outbred low food diet females *N* = 119, Inbred control males *N* = 124, Inbred control females *N* = 95, Inbred low food diet males *N* = 117, Inbred low food diet females *N* = 104

We found some evidence for catch-up growth in mosquitofish. Fish exposed to the low food diet took significantly longer to mature and although statistically they were significantly smaller at maturity, they were still very similar in size to control fish (see below). Females matured at a significantly larger size than males when on the control diet, but not when they were on the low food diet (i.e. sex × diet interaction, GLMM then run separately for each food treatment: Control diet *P* = 0.003, Low food diet *P* = 0.687, Table 1). Females took significantly longer to reach maturity than did males. Males on the low food diet matured on average 20 days later than those on the control diet, while females on the low food diet took 28 days longer to mature than those on the control diet. We did not find any statistically significant sex by diet interactions for time to, or size at maturity. On average, low diet treatment males matured at 98.5 % of the size of the average control diet male and females matured at 96.3 % of the size of the average control diet female (Tables 1 and 2, Fig. 2).

Finally, neither juvenile survival nor sex ratio at maturation was affected by diet (Table 2).

## Discussion

The effects of inbreeding are expected to be exacerbated in stressful environments [14]. We tested this hypothesis by rearing inbred and outbred mosquitofish in two different food treatments (i.e. a stressful environment — low food diet and a non-stressful environment — control diet) and measured their growth rate, size, age at maturity, and their ability to show compensatory growth and catch-up growth. Our results revealed (1) no evidence for inbreeding depression in either the benign or more stressful rearing environments, (2) some evidence for catch-up growth, and (3) no evidence for compensatory growth.

We found no evidence for inbreeding depression for any of the measured traits (i.e. growth rates, adult size, and age at maturity). One reason that is often posited for a lack of inbreeding depression is that the expression of deleterious alleles depends on the environment an animal experiences [14], including the conditions in which animals are raised [48]. For example, previous studies have shown effects on inbreeding in the presence of certain stressors (e.g. chemicals or desiccation), but not others (e.g. heat resistance; [49]). Others have found a modest correlation between the extent of inbreeding depression and the level of dietary stress [50–52]. Our low food diet lead to almost zero growth over a three-week period and is thus comparable to a very harsh natural environment. The fact that we did not find effects of inbreeding depression in either of our experimental treatments, especially given our large sample size (*N* = 908 fry), is thus robust evidence that the traits we measured do not suffer inbreeding depression in *Gambusia holbrooki* under the stressful conditions the fish experienced in this experiment (i.e. three weeks with insufficient food for juvenile growth). We have previously shown [38] that this diet reduces male attractiveness and is therefore, by definition, stressful (see [14]).

The presence and magnitude of inbreeding depression may differ depending on which life stages and/or traits are measured [53]. For example, some studies show no effect of inbreeding depression on body size, but do show an effect on time to development [7]. The traits we measured (i.e. growth, time to maturation, survival) are major life-history traits with large effects on fitness in many species [25, 54] that are therefore expected to be condition-dependent [55]. These traits should be particularly prone to inbreeding depression because condition is assumed to be affected by multiple loci across the genome [10], so this result was somewhat surprising. One explanation for a lack of inbreeding effect is that maternal and family effects on fitness might overshadow effects associated with inbreeding [39, 56] due to high variance among families [57]. We can dismiss this explanation, however, as we explicitly controlled for sire, dam, and family effects. Another explanation for a lack of inbreeding depression for the traits we measured is that mosquitofish have purged deleterious alleles for metabolic responses to low food availability as a result of periodic population bottlenecks [58, 59]. In support of this, previous studies looking at the effects of inbreeding depression on population size and population growth rate under two different salinities in mosquitofish did not find evidence for inbreeding depression [39]. However, in our population we have directly shown that lower heterozygosity in males (natural rather than experimental in origin) leads to significantly lower reproductive success (Head ML, Kahn AT, Keogh SJ, Jennions MD unpublished observations), suggesting that inbreeding *does* reduce fitness, but not because of its effects on adult size or growth rates.

We did not find any evidence of compensatory growth in our study. Fish in the stressful low food environment did not show faster growth rates after food restriction early in life compared to fish on the control diet. This result, contrasts with that of Livingston et al. [37] who found partial compensatory growth for females, but it agrees with their findings for males. Both studies used the same diet manipulation so the reasons for the difference are unclear. However, our findings are in accordance with the wider trend that fish generally show little evidence for compensatory growth compared to other taxa [30]. One reason that has been posited for this taxonomic difference is that ectotherms have indeterminate growth and are under less pressure to rapidly achieve a large final size than taxa with determinate growth. However, the evidence from mosquitofish does not support this explanation. Male mosquitofish have determinate growth but do not show compensatory growth (this study and [37]), while females have indeterminate growth but there is some evidence for compensatory growth ([37], but not our study). If we assume selection for large body size is comparable across the sexes (although this might not be the case in Poeciliids where smaller males could have a mating advantage: see [60] and Head ML, Kahn AT, Keogh SJ, Jennions MD unpublished observations) we would expect to see compensatory growth in males, but not females, if an explanation based on determinate versus indeterminate growth is correct.

Although we did not observe compensatory growth in response to food deprivation, fish in the low food diet did mature at a very similar (albeit statistically significantly smaller) size to those on the control diet because they delayed their maturation (i.e. ‘catch-up growth’ sensu [30]). Similar results have been found for another poeciliid fish the guppy (*Poecilia reticulata*) [61, 62]. In these studies, guppies showed a reduction in growth rate, an increase in age at maturity, and a decrease in size at maturity after a period of reduced food availability. Delaying maturation to achieve a larger adult size may be physiologically less costly than increasing growth rate [63], but it could still reduce lifetime reproductive success if it leads to less time in the breeding pool [64]. The relative magnitude of these two costs could be important in determining whether species compensate for restricted growth during development by increasing their subsequent growth or by delaying maturation.

## Conclusions

There was no interaction between inbreeding and diet restriction during development on juvenile survival, growth or size, and age at maturity. This indicates that these traits do not suffer from inbreeding depression, even after individuals are exposed to a seemingly stressful low food environment (see [38]). It implies that how mosquitofish respond to a restricted diet during early development does not depend on phenotypic quality (assuming inbred individuals are, at least for some traits, inferior due to their lower heterozygosity). Of course, our results do not rule out that inbreeding depression occurs in *G. holbrooki*, nor do they exclude a G × E interaction between inbreeding and rearing environment. Previous studies highlight that it is important to look at the effects of inbreeding over all life stages and for multiple traits [13]. Looking at only single life stages or a limited set of traits may under- or overestimate the effects of inbreeding because it does not take into account potential trade-offs between life stages or traits [11, 13, 65]. For example, in mosquitofish, males that have a poor start in life (i.e. reared on a restricted diet) are less attractive to females than those reared on a control diet in simple two-choice mate association tests [38]. This illustrates the potential for hidden long-term costs of a stressful environment. Furthermore, we reared fish individually (to reduce variation), but this eliminates any potential for reduced social competitiveness to affect growth and adult size. Perhaps most importantly, in a companion study we tested how the inbreeding status and diet treatment of males affect their ability to gain paternity when they compete for females in a socially competitive environment (Vega-Trejo, R, Head ML, Keogh SJ, Jennions MD unpublished observations). We found that inbred males are significantly less successful, but that there is no effect of diet, nor any interaction between diet and inbreeding on male reproductive success. This suggests that inbreeding *does* ultimately reduce fitness and perhaps overrides the effect seen in attractiveness due to diet [38]. The current study indicates, however, that this is not because inbreeding affects adult size or growth rates. The proximate basis of inbreeding depression in male *G. holbrooki* therefore remains to be determined. One possibility that we are currently testing is that inbreeding lowers sperm competitiveness.

## Methods

### Study system

Mosquitofish (*Gambusia holbrooki*) are small Poeciliid fish endemic to North America and introduced worldwide [66]. They are non-migratory and are often resident in relatively small bodies of water such as ponds and streams [67]. This makes it likely that inbreeding occurs naturally in situations where a few fish become isolated in a small area.

### Origin and maintenance of fish

Our laboratory stock of mosquitofish derives from 151 wild-caught gravid females (females mate multiply so broods have multiple sires) collected in Canberra, Australia in February and March 2013. This work was conducted under the ethic approval that was granted by ANU animal ethics protocol A2011/64. Collection permits were not required for this study as *G. holbrooki* are a pest species in Australia. F_1_ generation offspring were kept in single sex tanks under a 14:10 h photoperiod at 28 °C and fed *ad libitum* with *Artemia nauplii* and commercial flakes. Females were reared to adulthood and separated before sexual maturity to ensure virginity.

### Experimental design

The design to create inbred and outbred fish is fully described in Vega-Trejo et al. [46]. In brief, we set up 150 unique breeding pairs that were randomly created from F_1_ individuals (described above, avoiding any pairing of fish with the same mother). From these pairings we obtained 58 outbred F_2_ full-sib families with sufficient numbers of both sexes to be used in our experimental design. The design required two F_2_ families per block to create both inbred and outbred offspring (described below). We established 29 experimental blocks.

#### Inbred versus outbred fish

**Fig. 3:**
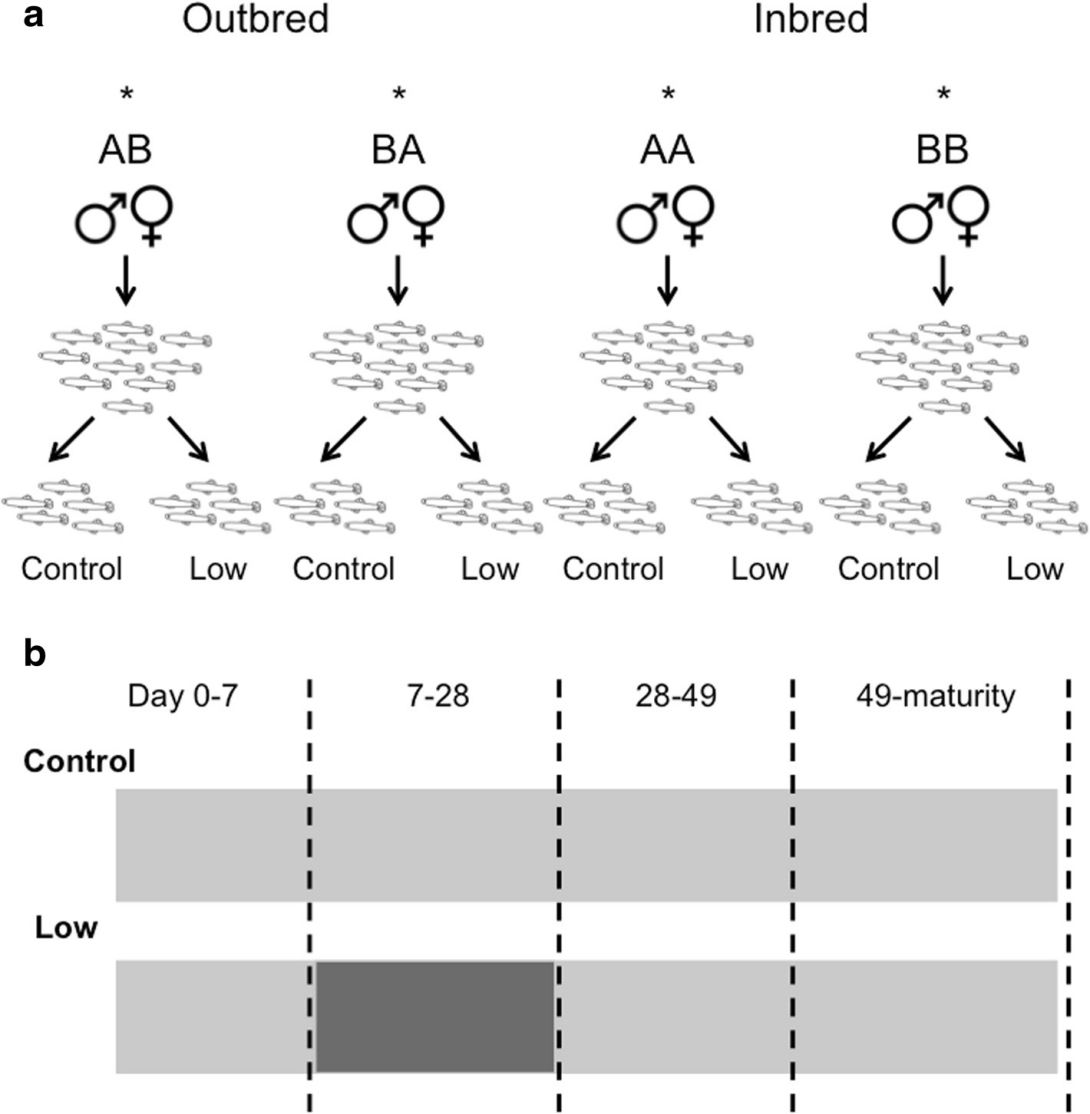
Schematic of experimental design. **a** Shows block design used to create outbred and inbred fish. For each block we set up 1-4 females per cross-type. Within each block the same potential number of females contributed to each cross-type. A single male contributed to each cross-type so that, within each block, the offspring of each cross-type were either full or paternal half-siblings. We ended up with 604 inbred offspring from 109 mothers and 54 fathers, and 617 outbred offspring from 128 mothers and 55 fathers. Offspring from each cross-type were evenly distributed across food treatments. * indicate matings, **b**) shows feeding regime for each diet treatment. Light shade indicates Ad lib food was given twice a day, dark shade indicates 3 mg of food every other day. Dashed lines indicate points at which measurements were taken

We used a fully balanced block design that involved mating individuals from two families (e.g. A and B). Brothers and sisters from full sibling families were paired to create inbred offspring (AA, BB) and outbred offspring with reciprocal crosses for each cross-type (BA, AB; Fig. 3). Males and females were placed together for 1 week to allow mating. Females were then placed in individual 1 L tanks and checked twice daily for babies over a six-week period. Those that had not given birth were re-introduced to the male for another 7 days to increase the number of offspring produced. We recorded gestation time, female standard length (SL = snout tip to base of caudal fin) and the number of offspring produced [46]. To measure female size, fish were anaesthetized by submersion in ice-cold water for a few seconds to reduce movement and then photographed alongside a microscopic ruler (0.1 mm gradation). We also recorded the size of offspring within 18 h of being born using images obtained after placing live fish into a square container (27 wide × 27 mm long × 22 high) containing water to a depth of 1 mm. Measurements were made using *Image J* software [68]. These, and all subsequent, size measures were made blind to treatment type (see [69]).

#### Diet

We raised a maximum of 10 fry from each cross-type, each reared individually in separate 1 L tanks. All fish were fed *ad libitum* with *A. nauplii* twice a day for seven days and then photographed for later measurements (as described above). Each fish was then randomly assigned to the control or low food diet at one week of age. Control diet fish continued being fed *ad libitum* with *A. nauplii* twice a day until the end of the experiment (*N* = 472). Fish in the low food diet had their food restricted from 7 to 28 days of age (i.e. experienced limited food availability for 21 days) when they were fed 3 mg of *A. nauplii* once every other day (less than 25 % of the amount of food; *N* = 492). From day 28 onward their diet was returned to the same level as that of control diet fish (Fig. 3). This low food diet minimises growth (see diet effect in Table 1, Fig. 1), but did not increase mortality (see [37]).

#### Size measurements

All fish were photographed (as for females above) on day 28 (end of low food diet) and again on day 49. Thereafter, fish were inspected three times per week to determine the time to maturity and photographed to obtain their SL once mature. Females were considered mature when yellow spots were evident in the abdomen, indicating yolked eggs [70]. Males were considered mature when their gonopodium (intromittent organ modified from the anal fin) was translucent, with a spine visible at the tip [70, 71]. All inspections for maturity were made blind to treatment. Unexpectedly (see [37]) some, mainly control fish (*N* = 133) matured before day 49 (68 outbred and 51 inbred on control diet; 8 outbred and 6 inbred on low diet). In our analyses we treat these fish are though they matured on day 49. In further sensitivity analyses we alternatively gave control diet individuals lower ages at maturity (between 28 and 49 days). This did not qualitatively alter our results, nor did analysing the effect of inbreeding based only on fish on the low diet treatment (results are not presented, but data is available in Dryad).

### Inbreeding and heterozygosity

If we treat the source population as a baseline of outbred individuals then *f* = 0.25 for the offspring of brother-sister matings.

We used RAD-tag to detect SNPS that provided us with data of genome wide heterozygosity based on 3045 SNPs from a subsample of 122 males (see Additional file 1 for full methods). We then quantified the proportion of loci per male that were heterozygous, and tested whether the mean level of heterozygosity differed between inbred and outbred males.

### Statistical analysis

#### Diet & inbreeding effects

We analysed the fixed effect of diet, inbreeding (inbred versus outbred), sex, and all possible two-way and three-way interactions using generalised linear mixed models (GLMM) in *R 3.0.2* software [72] with separate models for each response variable. We ran models for *size at birth, growth rates, size at maturity*, and *age at maturity* using a Gaussian error distribution. We also ran a model for *age at maturity* with a negative binomial distribution of the error due to the fairly high number of fish classified as maturing on day 49. Each model was fitted using the *lme4* package in *R 3.0.2* software with block, maternal identity, and sire identity as random factors. All size measurements were log transformed. All parameters estimated were tested for significance using Anova with Type III Wald chi-square tests. Model simplification (i.e. removing non-significant interaction terms) did not change our results. Figures are presented using raw data rather than model predictions unless otherwise indicated. We have previously reported the effects of inbreeding on birth size and growth to 7 days using a subset of the current data ([46]; the current data set includes offspring produced more than six weeks after initial pairing of fish).

#### Compensatory growth

There was no initial size difference at birth between inbred and outbred fish (see Results). Additionally, we tested whether inbreeding and/or sex affected growth to day 7 (i.e. the beginning of the diet treatment). Growth was always quantified as the instantaneous rate of growth, G = ln (L_t1_/L_t0_) / *t*, where L refers to the length (SL) at *t*_n_ age and *t* is time (day) of measurement. There was no difference in initial growth to day 7 between inbred and outbred fish (see Results). The fish assigned to the four categories (inbreeding × diet) were therefore the same mean size at the start of the diet treatment.

We tested for an effect of diet on growth while the treatment was applied by comparing the growth of control and low food diet fish between days 7 and 28. We then tested for an early compensatory growth response of low food diet fish by comparing growth when returned to the control diet. To account for a potential effect of a difference in size at the start of the relevant growth period (i.e. because growth slows with absolute size), we compared growth from days 7 – 28 for the control diet fish [ln (L_day 28_ / L_day7_) / 21] and days 28-49 for the low food diet fish [ln (L_day 49_ / L_day28_) / 21] because the mean size of fish in the two groups was very similar at the start of the respective growth periods (mean control diet fish day 7: 11.07 ± 0.03, mean low food diet fish day 28: 11.76 ± 0.04). Then we tested for an overall effect of compensatory growth by testing for a difference in the instantaneous growth rate for each fish from an age giving a comparable initial body size (day 7 for control diet fish, day 28 for low food diet fish) to maturation. The duration of this period varied among individuals within and among treatments due to the time taken to reach maturity. Finally, we tested for catch-up growth evidenced by differences in length and age at maturity.

We also tested for any effect of diet, inbreeding or sex on survival and the offspring sex ratio using models with a binomial distribution of the error. These models used only fish that reached maturity.

### Ethics

This work was conducted under the ANU animal ethics protocol, granted by animal use permit: ANU AEEC animal ethics protocol A2011/64. Collection permits were not required for this study as *G. holbrooki* are a pest species in Australia.

### Consent to publish

Not applicable.

### Availability of data and materials

Data is deposited in Dryad: doi:10.5061/dryad.mb2gb.

## Additional file

Additional file 1: Provides the methods used to obtain data of genome wide heterozygosity. (DOCX 141 kb)
