# Supplementary material for: Inbreeding depression does not increase after exposure to a stressful environment: a test using compensatory growth
Source: BMC Evol Biol. 2016 Apr 1;16:68. doi: 10.1186/s12862-016-0640-1 (PMC4818490; doi:10.1186/s12862-016-0640-1)
Supplement: Additional file 1: — Provides the methods used to obtain data of genome wide heterozygosity. (DOCX 141 kb) [file 12862_2016_640_MOESM1_ESM.docx]

**Additional file 1**

To determine heterozygosity for the fish in our experiment we took tissue samples from a subsample of males (n= 122). DNA was extracted from the tail muscle/caudal fin using Qiagen DNeasy Blood and Tissue Kits following the manufacturer’s instructions. After extraction DNA samples were sent to the commercial genotyping service Diversity Arrays. This company has developed a widely used technique called DArTseq™. DArTseq™ represents a combination of DArT complexity reduction methods and next generation sequencing platforms [1-4]. It is a new implementation of sequencing complexity reduced representations [5] and more recent applications of this concept on next generation sequencing platforms [6, 7]. The technology is optimized for each organism by selecting the most appropriate complexity reduction method based on both the size of the representation and the fraction of a genome selected for assays. Four methods of complexity reduction were tested in *Gambusia* (data not presented) and the PstI-HpaII method was selected. DNA samples were processed in digestion/ligation reactions principally as per [3] but replacing a single PstI-compatible adaptor with two different adaptors corresponding to two different Restriction Enzyme (RE) overhangs. The PstI-compatible adapter was designed to include Illumina flowcell attachment sequence sequencing primer sequence and “staggered” varying length barcode region similar to the sequence reported by [7]. Reverse adapter contained flowcell attachment region and HpaII-compatible overhang sequence. Only “mixed fragments” (PstI-HpaII) were effectively amplified in 30 rounds of PCR using the following reaction conditions: 1. 94° C for 1 min; 2. 30 cycles of 94° C for 20 sec 58° C for 30 sec 72° C for 45 sec; 3. 72° C for 7 min. After PCR equimolar amounts of amplification products from each sample of the 96-well microtiter plate were bulked and applied to c-Bot (Illumina) bridge PCR followed by sequencing on Illumina Hiseq2500. The sequencing (single read) was run for 77 cycles.

Sequences generated from each lane were processed using proprietary DArT analytical pipelines. In the primary pipeline the fastq files were processed to filter away poor quality sequences applying more stringent selection criteria to the barcode region than the rest of the sequence. In that way the assignments of the sequences to specific samples carried in the “barcode split” step are very reliable. Approximately 2500000 (+/- 7%) sequences per barcode/sample were used in marker calling in routine DArTseq assay but we applied a more cost effective version of the assay using half of the normal tag number (average of 1.3 million per sample). Finally identical sequences were collapsed into “fastqcall files”. These files were used in the secondary pipeline for DArT PL’s proprietary SNP and SilicoDArT (presence/absence of restriction fragments in representation) calling algorithms (DArTsoft14). This clean-up process resulted in a comprehensive data set of approximately 3045 SNPs with an average call rate of 97.7% and a reproducibility rate of 99.3%.

*Heterozygosity*

We estimated heterozygosity by using the number of markers that were scored as heterozygous divided by the total number of successfully classified markers for that fish.

**Supplementary References**

1. Courtois B, Audebert A, Dardou A, Roques S, Ghneim-Herrera T, Droc G, Frouin J, Rouan L, Goze E, Kilian A *et al*: **Genome-Wide Association Mapping of Root Traits in a Japonica Rice Panel**. *Plos One* 2013, **8**(11).

2. Cruz VMV, Kilian A, Dierig DA: **Development of DArT Marker Platforms and Genetic Diversity Assessment of the US Collection of the New Oilseed Crop Lesquerella and Related Species**. *Plos One* 2013, **8**(5).

3. Kilian A, Wenzl P, Huttner E, Carling J, Xia L, Blois H, Caig V, Heller-Uszynska K, Jaccoud D, Hopper C *et al*: **Diversity arrays technology: a generic genome profiling technology on open platforms**. *Methods in molecular biology (Clifton, NJ)* 2012, **888**:67-89.

4. Raman H, Raman R, Kilian A, Detering F, Carling J, Coombes N, Diffey S, Kadkol G, Edwards D, McCully M *et al*: **Genome-Wide Delineation of Natural Variation for Pod Shatter Resistance in Brassica napus**. *Plos One* 2014, **9**(7).

5. Altshuler D, Pollara VJ, Cowles CR, Van Etten WJ, Baldwin J, Linton L, Lander ES: **An SNP map of the human genome generated by reduced representation shotgun sequencing**. *Nature* 2000, **407**(6803):513-516.

6. Baird NA, Etter PD, Atwood TS, Currey MC, Shiver AL, Lewis ZA, Selker EU, Cresko WA, Johnson EA: **Rapid SNP Discovery and Genetic Mapping Using Sequenced RAD Markers**. *Plos One* 2008, **3**(10).

7. Elshire RJ, Glaubitz JC, Sun Q, Poland JA, Kawamoto K, Buckler ES, Mitchell SE: **A Robust, Simple Genotyping-by-Sequencing (GBS) Approach for High Diversity Species**. *Plos One* 2011, **6**(5).
